# Special Issue: Application of SERS for Nanomaterials

**DOI:** 10.3390/nano11123300

**Published:** 2021-12-06

**Authors:** Ronald L. Birke

**Affiliations:** Department of Chemistry and Biochemistry, The City College of the City University of New York, 160 Convent Avenue, New York, NY 10031, USA; rbirke@ccny.cuny.edu

Surface-enhanced Raman scattering (SERS) is now a relatively mature field of spectroscopy, with it having been almost 50 years since its first experimental demonstration [1]. A main feature of SERS is the requirement for a surface substrate that facilitates the enhancement mechanism. This surface-enhancing substrate can produce a plasmon resonance electromagnetic enhancement of the stimulating light and/or facilitate a photon driven charge-transfer (CT) resonance [2,3]. The combined plasmonic and CT resonance process occurs on substrates of metal nanoparticles (NPs) such as Ag, Au, Cu, and other metals [4], whereas a photon driven charge-transfer resonance (CT-SERS) is the main enhancing process on semiconductor nanoparticles [5]. Within the variety of enhancement processes possible, an additional scattering mechanism involves a combination of the plasmonic resonance with a molecular resonance sometimes called surface-enhanced resonance Raman scattering (SERRS) [6]. The molecular resonance generates Franck–Condon scattering while the charge transfer resonance generates Herzberg–Teller scattering [2,3,7]. This diversity of scattering mechanisms makes the formulation of a surface Raman theory, which covers all cases, challenging. The various enhancement mechanisms, all involving engineered or assembled nanoparticle surfaces, can boost the relatively low normal Raman signal to substantial and possibly “giant” intensities, even allowing for single-molecule detection in the most efficient cases [8]. Furthermore, the rich Raman vibrational spectra obtained with SERS methods provide molecular level information and chemical identity. These properties have established SERS methodologies as ultra-sensitive techniques for analytical sensing of chemical and biochemical compounds and for the investigation of the properties of nanostructured plasmonic metal [9] and semiconductor dielectric substrates [10,11]. Since the sensing process is linked to progress in the design and fabrication of new nanomaterial surfaces, a recent Special Issue of this journal was specifically devoted to this aspect of SERS development [12]. However, SERS is still being developed in terms of the fabrication of new nanomaterial surface substrates, new applications in chemical and biochemical sensing, improvement of methods for interrogating surface structure such as imaging with tip-enhanced Raman scattering (TERS), studies of chemical reactions with SERS, and the computational simulation of the surface Raman scattering spectra at different nanomaterial interfaces. The present Special Issue includes research papers which address these areas of SERS developments.

There are eight research papers in this Special Issue which explore important developments for future applications of SERS. The first paper by Pei Dai et al. [13] illustrates the fabrication of inexpensive hydrophobic pure Cu chips and those modified with a thin shell of Ag. The chips are made by depositing the Cu nanoparticles on a flexible fabric support by chemical reduction methods with the Ag layer formed by a replacement method. The Ag-Cu chip substrates show a higher enhancement factor with good stability, maintaining 80% of its intensity for up to two months. Several organic molecules are investigated and separated into two groups based on their HOMO/LUMO levels with respect to the equilibrated Fermi level of Cu-Ag and the possibility of photoinduced charge transfer, PICT. Additionally, there are three papers involving the charge transfer (CT) mechanism for wide-band gap semiconductors-ZrO_2_ [14], TiO_2_ [15], and MoO_3_ [16]. Yi et al. [14] investigated two crystal forms of ZrO_2_ as substrates for SERS. Raman spectral shifts indicate that the scattering mechanism is a CT mechanism. Using hydrothermal synthesis with additives for selective growth, they show that the 99.7% tetragonal phase is superior to the monoclinic phase since it has highest degree of charge transfer parameter. The energy levels of surface defect states in the band gap of ZrO_2_ play a decisive role in rationalizing the CT scattering mechanism from ZrO_2_ to the LUMO of 4-mercaptobenzoic acid (4–MBA). Birke and Lombardi [15] use Density Functional Theory (DFT) to simulate the surface Raman spectra of the solar cell dye N3 adsorbed on a Ti_5_O_10_ nanoparticle cluster model. This small TiO_2_ cluster was found to have similar properties to clusters as large as Ti_38_O_76_. Enhancement factors of about 1 × 10^3^ and 2 × 10^2^ for resonance Raman and charge transfer surface scattering mechanisms, respectively, were found from the simulations. The CT-SERS simulation shows that a direct dye to semiconductor photoinduced electron transfer is possible without going through an intermediate dye excited state, which is relevant to the excitation mechanism of the dye-sensitized solar cell (DSSC). In the paper by Chu et al. [16], a composite system is fabricated by co-sputtered Ag/MoO_3_ on polystyrene microspheres which contains abundant oxygen vacancy defects. 4-Aminothiophenol (PATP) is used as a SERS probe molecule and the oxygen vacancy defects in MO_3_ are indicated as intermediate energy channels for electron transport between the Fermi level of Ag and the LUMO of PATP. Another study in this issue of a composite system by Lin Guo et al. [17] utilizes 4-mercaptobenzoic acid (MBA) as a bridge between Au nanorods (NRs) of different length to diameter ratios (L/D) and a Cu_2_O semiconductor with a consistent 15 nm thickness in a core–shell (Au NR–MBA@Cu_2_O) structure. Here, the sulfur end of the molecule binds to the Au atom of the nanorod and the carboxylate end binds to Cu^+^ of the Cu_2_O shell. The L/D ratios of the NRs adjust the surface plasmon resonance (SPR) and the specific surface area of the nanorods. These assemblies are investigated for plasmon absorption characteristics, changes in SERS bands, and for the degree of charge transfer as a function of L/D ratio. The CT takes place from the Au NRs through the LUMO level of MBA to the conduction band (CB) of the Cu_2_O shell. When the wavelength of the SERS incident laser light is close to the maximum of the longitudinal SPR absorption curve, the degree of the charge transfer process increases. Furthermore, increased surface area of Au NRs correlates with the movement of CB of Cu_2_O closer to the LUMO level of MBA facilitating the degree of charge transfer. These studies show the value of SERS in elucidating the basic photo-physics in nanomaterial systems.

The method of TERS allows for imaging below the Abbe half-wavelength diffraction limit of the imaging light and can show resolutions in the tens of nanometers. This method has a significant future for revealing new chemistry and physics in nanostructured systems. The paper of Mandelbaum et al. [18] explores the effect of shape (hemisphere, hemispheroid, ellipsoidal cavity, ellipsoidal rod, nano-cone) and material (Ag, Au, Al) on surface enhancement using the finite element numerical and analytical approximation methods for single tip structures, a four-probe configuration, and for pixel arrays of tips. The hemispheroid was recommended as the best geometry for the nanoparticle tips.

The remaining two articles in this Special Issue [19,20] show noteworthy applications of SERS for biochemical and chemical sensing. Zavyalova et al. [19] develop a simple rapid quantitative colloidal Ag based SERS method for the detection of the COVID-19 coronavirus SARS-CoV-2. The selectivity is obtained with the DNA RBD-1C aptamer which selectively binds the receptor binding domain (RBD) of the surface S-protein of the virus, and the detection sensitivity comes from the Bodipy Fl SERS reporter molecule. The essence of this assay is that the SERS intensity increases in the presence of increasing SARS-CoV-2 but decreases with increasing non-specific viral particles. The method is one-step and fast (7 min), with a high sensitivity (LOD of 5.5 Å~10^4^ TCID50/mL). In the final article in this Special Issue, Xu et al. [20] studies the SERS detection methodology for the notorious herbicide and crop desiccant ‘glyphosate’ (GLP) or N-(phosphonomethyl)gycine. This compound is the active ingredient in weed killers such as ‘Roundup’ and has been claimed to cause non-Hodgkin’s lymphoma. An isotopic glyphosate denoted 13-GLP with ^13^C substitution at the 2-position, 2-^13^C-glyphosate, as-well-as the non-isotopic 12-GLP are investigated. The method is based on the ninhydrin reaction with the amino group of GLP catalyzed by MoO_4_ to form a purple color dye product, PD. The PD products formed from both 12-GLP and 13-GLP are studied. Raman and Fourier transform infrared (FTIR) spectra are obtained in the absence of Ag nanoparticles (NPs) and SERS spectra are obtained in the presence of Ag NPs. DFT calculations at the B3LYP/6-311++G(d,p) level are used to interpret the vibrational band assignments of the spectra. The use of isotopic substitution is recommended as a promising area for future use in the interpretation of SERS spectra.

## Data Availability

Not applicable.
